# COMT Val^158^Met Polymorphism Exerts Sex-Dependent Effects on fMRI Measures of Brain Function

**DOI:** 10.3389/fnhum.2017.00578

**Published:** 2017-12-06

**Authors:** Amanda Elton, Christopher T. Smith, Michael H. Parrish, Charlotte A. Boettiger

**Affiliations:** ^1^Department of Psychology and Neuroscience, University of North Carolina, Chapel Hill, NC, United States; ^2^Bowles Center for Alcohol Studies, University of North Carolina, Chapel Hill, NC, United States; ^3^Neuroscience Curriculum, University of North Carolina, Chapel Hill, NC, United States; ^4^Biomedical Research Imaging Center, University of North Carolina, Chapel Hill, NC, United States

**Keywords:** fMRI, dopamine, COMT, delay discounting, sex differences

## Abstract

Evidence suggests that dopamine levels in the prefrontal cortex (PFC) modulate executive functions. A key regulator of PFC dopamine is catechol-*O*-methyltransferase (COMT). The activity level of the COMT enzyme are influenced by sex and the Val^158^Met polymorphism (rs4680) of the *COMT* gene, with male sex and Val alleles both being associated with higher bulk enzyme activity, and presumably lower PFC dopamine. *COMT* genotype has not only been associated with individual differences in frontal dopamine-mediated behaviors, but also with variations in neuroimaging measures of brain activity and functional connectivity. In this study, we investigated whether *COMT* genotype predicts individual differences in neural activity and connectivity, and whether such effects are sex-dependent. We tested 93 healthy adults (48 females), genotyped for the Val^158^Met polymorphism, in a delay discounting task and at rest during fMRI. Delay discounting behavior was predicted by an interaction of *COMT* genotype and sex, consistent with a U-shaped relationship with enzyme activity. *COMT* genotype and sex similarly exhibited U-shaped relationships with individual differences in neural activation, particularly among networks that were most engaged by the task, including the default-mode network. Effects of *COMT* genotype and sex on functional connectivity during rest were also U-shaped. In contrast, flexible reorganization of network connections across task conditions varied linearly with *COMT* among both sexes. These data provide insight into the potential influences of COMT-regulated variations in catecholamine levels on brain function, which may represent endophenotypes for disorders of impulsivity.

## Introduction

The catechol-*O*-methyltransferase (COMT) enzyme is responsible for degrading the majority of prefrontal cortical dopamine. The gene encoding COMT (*COMT*) has a common functional polymorphism, a valine (*Val*) to methionine (*Met*) substitution (Val^158^Met, rs4680), which is associated with substantially reduced COMT activity (Lachman et al., 1996). Thus, activity levels of the COMT enzyme vary according to genotype (i.e., *Met/Met* < *Val/Met* < *Val/Val*). Because variations in COMT enzyme activity levels are presumed to produce corresponding alterations of frontal dopamine levels (Gogos et al., 1998), this genotype is hypothesized to influence extracellular dopamine levels in the frontal cortex in an allele dose-dependent manner (i.e., *Val/Val* < *Val/Met* < *Met/Met*).

Consistent with the role of the COMT enzyme in prefrontal catecholamine degradation, numerous studies have linked this *COMT* functional polymorphism to individual differences in prefrontal-mediated functions. For example, *COMT* genotype has been associated with a variety of executive behaviors including delay discounting (Boettiger et al., 2007; Paloyelis et al., 2010; Smith and Boettiger, 2012), working memory (Bruder et al., 2005), and set shifting (Malhotra et al., 2002; Tunbridge et al., 2004). Corresponding effects of *COMT* genotype on behavioral task-related neural activity measured with functional magnetic resonance imaging (fMRI) have been demonstrated (Smolka et al., 2005; Winterer et al., 2006; Boettiger et al., 2007; Congdon et al., 2009; de Frias et al., 2010; Stokes et al., 2011), often with large effect sizes (Mier et al., 2010). *COMT* genotype has more recently been associated with brain functional connectivity at rest (Tian et al., 2013; Tunbridge et al., 2013; Markett et al., 2016). Thus, this polymorphism has been extensively linked to individual differences in executive behaviors and with associated functional neuroimaging measures, which is often attributed to its influence on prefrontal dopamine.

Intermediate levels of dopamine are deemed optimal for many executive functions. Reflecting its presumed influence on prefrontal dopamine levels (Cools and Robbins, 2004), effects of the *COMT* Val^158^Met polymorphism frequently exhibit U-shaped or inverted U-shaped relationships (Vijayraghavan et al., 2007; Smith and Boettiger, 2012). Although the direction of *COMT* genotype effects is sometimes inconsistent across studies (Mier et al., 2010), perhaps due to differences in the “optimal” dopamine levels for different behaviors (Stein et al., 2006), discrepancies in *COMT* effects may also occur when individuals are shifted along the U-shaped curve due to state or trait variations in dopamine. For example, effects of this genotype are proposed to depend on other modulators of dopamine including stress (Stein et al., 2006) or task performance (Jacobs and D'Esposito, 2011), which result in apparent shifts of the observed relationships along a U-shaped curve. Interactions of *COMT* Val^158^Met genotype with other genetic polymorphisms that regulate dopamine signaling also produce inverted U-shaped effects corresponding with the number of dopamine-promoting alleles (Yacubian et al., 2007; Tian et al., 2013). Furthermore, the relationship between *COMT* genotype and impulsive decision-making behavior in a delay discounting task are modulated by age, producing a U-shaped relationship with both genotype and putative age-related declines in frontal dopamine levels (Smith and Boettiger, 2012). Similar inverted U-shaped effects across genotype and age have been noted for functional connectivity of the medial frontal cortex at rest (Meyer et al., 2016), indicating that *COMT* effects persist in the absence of any particular behavior.

Another major moderator of *COMT* effects is sex. Sex and sex steroids represent significant sources of variation in COMT enzyme activity whereby COMT enzyme activity is reduced in women compared with men (Chen et al., 2004), likely due to suppression by estradiol (Cohn and Axelrod, 1971). In fact, the size of the effect of sex on COMT enzyme activity is comparable to the size of the effect of each *Met* allele (Cohn and Axelrod, 1971). This sex-related modulation of COMT enzyme activity, coupled with estradiol-induced increases in frontal dopamine signaling (Jacobs and D'Esposito, 2011; Schendzielorz et al., 2011; Smith et al., 2014a), supports the contention that females generally have greater levels of prefrontal dopamine than males (Jacobs and D'Esposito, 2011). Thus, whereas V*al/Val males* would generally be predicted to have the lowest dopamine levels, *Met/Met* females of similar age would have the highest levels. However, given the complex effects of sex and sex steroids on both COMT enzyme activity and dopamine levels, the precise order of effects of sex and *COMT* genotype on dopamine levels may not strictly correspond with those on COMT enzyme activity (Chen et al., 2004). Regardless, the impact of these sex-dependent variations in COMT enzyme activity on measures of brain function and behavior have received little consideration to date.

Although a few studies have reported sex differences in *COMT* genotype effects on behavior, they are often restricted to reports of significant effects in one sex but not the other (Harrison and Tunbridge, 2008), with only limited evidence of U-shaped effects (Lang et al., 2007; Chen et al., 2011). Nonetheless, like *COMT* genotype, sex and sex steroids influence prefrontal-mediated behaviors (Bobova et al., 2009; Jacobs and D'Esposito, 2011; Peper et al., 2013; Smith et al., 2014a), and mounting evidence suggests that effects of *COMT* genotype differ between males and females (Gogos et al., 1998; Rybakowski et al., 2006; Lang et al., 2007; Harrison and Tunbridge, 2008; Chen et al., 2011; Jacobs and D'Esposito, 2011; Laatikainen et al., 2013). For example, sexually-dimorphic effects of *COMT* genotype have been reported for set-shifting (Rybakowski et al., 2006) and sensation-seeking personality (Lang et al., 2007). However, no study to our knowledge has examined the potential moderating influences of sex on *COMT* genotype in the context of delay discounting nor in the context of neuroimaging. Such an investigation of sex-dependent influences of *COMT* may shed light on the neurobiology of the sex differences present in many psychiatric disorders (Harrison and Tunbridge, 2008).

Here we investigate the hypothesis that *COMT* Val^158^Met genotype interacts with sex to affect certain measures of behavior and neural function according to a U-shaped relationship. Given the strong prior associations of this *COMT* polymorphism with prefrontal-mediated behaviors, and particularly for intertemporal decision-making (Paloyelis et al., 2010; Smith and Boettiger, 2012) and its neural correlates (Boettiger et al., 2007), we focused our investigation on data from an fMRI delay-discounting task. Rather than limiting our investigation to a specific brain region or network, we examined *COMT* genotype effects across multiple large-scale functional neural networks. Using fMRI, we scanned 93 healthy men and women both during a resting state and while performing a delay discounting task in which they made four different categories of decisions. We considered the effects of *COMT Val*^158^*Met* genotype and sex at multiple levels of function, including behavior, neural activation, neural connectivity, and neural flexibility. We report significant interacting effects of sex and genotype, producing U-shaped relationships between putative frontal dopamine and delay discounting behavior, neural activations during delay discounting, and functional connectivity and functional organization during the resting state. In contrast, effects of *COMT* genotype on a measure of functional connectivity flexibility were not U-shaped. The results demonstrate novel associations of *COMT* genotype and sex on multiple large-scale neural networks and provide further evidence that neurocognitive effects of this genotype may extend beyond its role in frontal dopamine regulation.

## Materials and methods

### Subjects

Healthy adults (*n* = 93; 48 females) aged 18–40 years (mean = 25.9 years) provided informed consent to participate in this study, which was approved by the University of North Carolina, Chapel Hill (UNC) Office of Human Research Ethics. Right-handedness, English as a native language, and at least a high school education (mean years of education = 16.3 ± 2.5) were required for study inclusion. Study exclusion criteria included psychiatric disorders based a structured clinical interview using DSM-IV criteria (Sheehan et al., 1998), known neurological disorders, or current psychoactive drug use (including medication but excluding moderate alcohol or caffeine intake). A urinalysis drug screen and breathalyzer test for recent alcohol consumption conducted on the day of the scan confirmed sobriety.

### Genotyping

All participants provided a saliva sample for genotyping (DNA Genotek, Kanata, Ontario, Canada). Participants were genotyped for the *COMT* Val^158^Met polymorphism (rs4680) using TaqMan technology (Applied Biosystems, Foster City, CA), as previously described (Smith and Boettiger, 2012; Kelm and Boettiger, 2013; Smith et al., 2014a,b, 2015; Swift-Scanlan et al., 2014). Participant genotypes comprised 20 *Val/Val* (12 males, 8 females), 48 *Val/Met* (20 males, 28 females), and 25 *Met/Met* (13 males, 12 females). Allele frequencies in this sample did not deviate from Hardy–Weinberg equilibrium (χ^2^ = 0.12, *df* = 1, *p* > 0.05). Participants were tested blind to genotype.

### Delay discounting task

While in the MRI scanner, participants performed a delay discounting task optimized for fMRI studies. The details of this task, in which participants selected between sets of smaller, immediate hypothetical monetary rewards (“*Now*”), or larger, delayed rewards (“*Later*”), have been reported previously (Boettiger et al., 2007, 2009; Altamirano et al., 2011; Smith and Boettiger, 2012; Kelm and Boettiger, 2013; Smith et al., 2014a, 2015; Elton et al., 2017) and are further detailed in the Supplementary Materials. Subjects underwent six consecutive functional MRI scans (~8 min per scan) each of which included 32 *Now*-vs.-*Later* decisions, consisting of four decision trial types presented psuedorandomly: “WANT,” “DON'T WANT,” “SOONER,” or “LARGER.”

### Behavior

To examine behavioral effects of *COMT* genotype, an impulsive choice ratio (ICR, Mitchell et al., 2005) was calculated as the proportion of *Now* choices (i.e., selection of the smaller, immediate reward) relative to all choices made during WANT trials:

$$ICR\;=\frac{Now}{Now+Later}$$

Greater discounting of delayed rewards corresponds with higher ICR values.

### Magnetic resonance imaging (MRI) data acquisition

For both resting-state and task fMRI scans, T2^*^-weighted echo-planar images (EPI) were acquired on a Siemens 3T Tim Trio MRI scanner with a 32-channel head coil using the following parameters: TR = 2,000 ms, *TE* = 25 ms, flip angle = 50°, 35 slices tilted 30° from the horizontal plane; FoV = 192 × 192 mm; voxel size = 3 × 3 × 4 mm with a 0.5 mm gap, matrix = 64 × 64. The resting-state scan (*n* = 1) and each task scan (*n* = 6) included 243 frames (~8 min). For the resting-state scan, participants viewed a white fixation cross on a black screen. The delay discounting task was displayed using E-Prime-2 software (Psychology Software Tools, Pittsburg, PA), which recorded participants' responses on an MRI-compatible button box.

T1-weighted high-resolution magnetization-prepared rapid gradient-echo (MPRAGE) structural images were also acquired for alignment and tissue segmentation purposes using the following parameters: *TR* = 2,530 ms *TE* = 2.27 ms, flip angle = 9°, matrix = 176 × 512, 512 slices, final resolution = 1 × 0.5 × 0.5 mm^3^.

### MRI data preprocessing

Functional image preprocessing consisted of the following steps completed in Analysis of Functional Neuroimages (AFNI Cox, 1996) software (v.16.0.1): correction for slice acquisition time, reorienting of oblique slices to the axial plane, image realignment for motion correction, despiking of noise time points, warping of functional images to a Montreal Neurological Institute (MNI) template, nuisance variable regression (white matter and cerebral spinal fluid signals as well as six motion covariates), linear detrending, spatial smoothing with an 8 mm full-width half-maximum Gaussian kernel, and scaling to percent signal change. Time points with high levels of artefactual signal were detected from a combination of head motion calculations and global signal intensity measures using The Artifact Detection Tools toolbox (ART; http://www.nitrc.org/projects/artifact_detect), which produced a set of corresponding regressors of no interest for activation and connectivity analyses.

### Regions-of-interest

**Figure 1:**
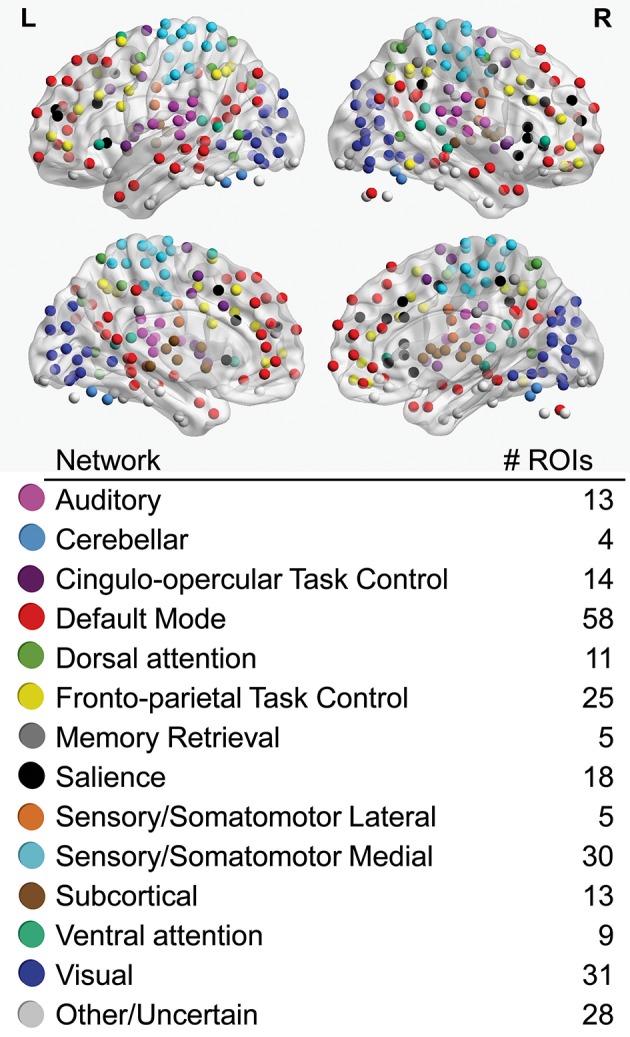
Regions-of-interest (ROIs), color-coded by association with large-scale networks, are visualized on left (L) and right (R) lateral (upper) and medial (lower) cerebral hemispheres. The total number of ROIs associated with each large-scale network is noted.

Regions-of-interest (ROIs) used in all analyses were 6 mm spherical volumes centered on 264 coordinates identified by Power and colleagues as functionally important nodes attributed to 13 large-scale functional networks (Power et al., 2011; Figure 1), including auditory (AU), cerebellar (CE), cingulo-opercular task control (CO), default mode (DM), dorsal attention (DA), fronto-parietal task control (FP), memory retrieval (ME), salience (SA), lateral sensorimotor (SML), medial sensorimotor (SMM), subcortical (SC), ventral attention (VA), and visual (VI) networks. To obtain a representative time series for each ROI, the first principle component time series was extracted from voxels within the ROI using AFNI's 3dmaskSVD after applying a high-pass filter (0.008 Hz) for task scans and both high-pass and low-pass filters for the resting-state scan (0.008–0.1 Hz).

### Task-related neural activations

Task-related activation for each ROI was defined by a contrast isolating subjective decision-making processes: WANT - (0.5 × SOONER + 0.5 × LARGER). Decision type cues were modeled as delta functions and decision-making trials were modeled as short epochs with a duration equal to the participant's response time for a given trial. The task design was convolved with a canonical hemodynamic response function in Statistical Parametric Mapping (SPM8) software. Additionally, we modeled six directions of head motion and covariates for outlier time points (ART toolbox), in addition to zero, first, and second order polynomial regressors for each task run. The model was fit with restricted maximum likelihood estimation in 3dREMLfit in AFNI.

### Resting-state functional connectivity

For the resting-state data, pairwise Pearson correlations between ROI time series were calculated and Fisher z-transformed to normalize the distribution, producing a 264 × 264 correlation matrix. Seven participants with missing resting-state data were not included in resting-state analyses, leaving 20 Val/Val (12 males, 8 females), 45 Val/Met (18 males, 27 females), and 21 Met/Met (11 males, 10 females).

Several graph theory indices were calculated to measure the functional organization of the brain during the resting-state, including functional connectivity strength, clustering coefficient, betweenness centrality, modularity (Newman's Q), local efficiency, and global efficiency. Functional connectivity strength was defined as the mean of the absolute value of the entire weighted graph. Using the Brain Connectivity Toolbox for Matlab, all other graph indices were calculated on binary, undirected graphs, and were averaged from a series of adjacency matrices of positive connections with densities between 5 and 30% at 1% intervals. Indices calculated at the node level (i.e., clustering coefficient, betweenness centrality, and local efficiency) were averaged across all 264 nodes to provide a single, whole-brain measure.

### Functional connectivity flexibility

Beta-series analysis enabled the calculation of task-related functional connectivity between ROIs during each of the four decision types (Rissman et al., 2004). Task design matrices were consistent with those described for task activations (above), with the exception that each decision-making trial was modeled as a separate regressor. The model was fit with restricted maximum likelihood estimation in 3dREMLfit in AFNI. Beta estimates for each of the four decision trial types (i.e., WANT, DON'T WANT, SOONER, LATER) were concatenated for each ROI. Next the pairwise Pearson correlations between ROIs were calculated for each decision trial type, producing four correlation matrices, which were Fisher z-transformed.

To calculate the extent to which functional connections reorganize between the five states (resting and four task decision types), functional connectivity flexibility was calculated as the Euclidean distance between each pair of correlation matrices, averaged across states for each ROI. For the 7 participants missing resting-state data, this measure only considered the four task decision states, and a covariate was included to account for this difference in statistical analyses.

### Statistical testing of COMT effects

Statistical tests of the effects of *COMT* genotype were estimated in linear models with the GLIMMIX procedure in SAS 9.4 (SAS Institute Inc., Cary, NC, USA). Genotype was coded as the number of *Met* alleles (*Val/Val* = 0, *Val/Met* = 1, *Met/Met* = 2) and sex was coded as 0 (males) or 1 (females). For network-based analyses of activation and functional connectivity, we estimated multilevel linear mixed models to account for the nested nature of the data, since each network includes multiple ROIs. These models were tested separately for each network (or pairs of networks for functional connectivity analyses) and, to account for variability of effects across network ROIs, included random slopes and intercepts for each ROI (or pairs of ROIs for functional connectivity analyses). In all models, *COMT* genotype effects covaried for age, socioeconomic status, menstrual cycle phase (categorically-defined to include women in cycle days 1–7, 8–15, and 18–30), and current hormonal birth control usage (binary). Analyses of resting-state functional connectivity also covaried for the average framewise displacement during the scan (Power et al., 2012) to control for apparent connections arising from head motion. Functional connectivity flexibility analyses similarly covaried for average framewise motion across the resting-state and six task scans. Each set of network-level results underwent false discovery rate (FDR) correction for 13 network comparisons.

## Results

### Behavioral data

**Figure 2:**
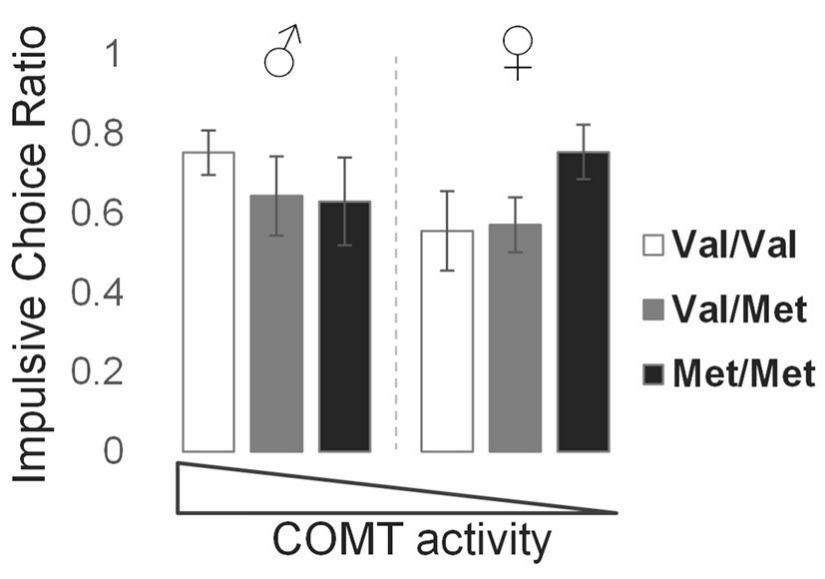
Effects of *COMT* genotype and sex on impulsive choice during a delay discounting task. Bar graphs represent the mean and standard error of impulsive choice ratio (ICR) values for each group, in order of theoretical frontal dopamine levels.

The mean (±SD) ICR value for this sample was 0.63 ± 0.32 (males: 0.67 ± 0.29; females: 0.59 ± 0.35). Due to non-normal distributions of raw ICR values, we performed an arcsine-root transformation prior to parametric testing. There was a significant genotype × sex interaction effect on ICR (*t* = −2.50, *p* = 0.014), producing a U-shaped relationship in which genotypes encoding the lowest and highest frontal dopamine levels, represented by *Val*/*Val* males and *Met*/*Met* females, respectively, demonstrated higher ICR values relative to genotypes encoding intermediate levels of frontal dopamine (Figure 2). These behavioral findings, together with the key neuromodulatory role of dopamine, led us then to examine sex and *COMT* genotype effects on brain network function, detailed in the following sections.

### Task-related neural activations

**Figure 3:**
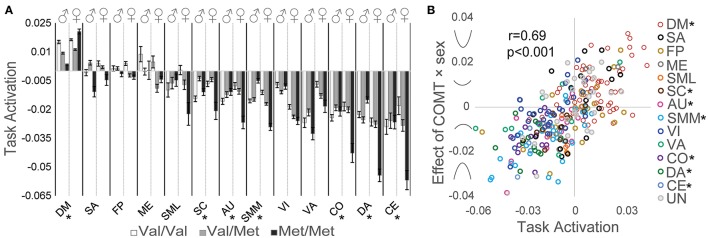
Effects of *COMT* genotype and sex on network activation during a delay discounting task. **(A)** Activation of each network is plotted by *COMT* genotype and sex. For display purposes, beta estimates of task-related activation for all ROIs of a given network are averaged. Error bars reflect standard errors of the mean. **(B)** Scatter plot of task activation effects plotted against *COMT* genotype × sex interaction effects on each of the 264 ROIs, color coded by association with large-scale networks. Symbols on the y-axis correspond with the direction of interaction effects, producing u-shaped (positive values) or inverted u-shaped (negative values) relationships. (^*^) Denotes statistically significant *COMT* genotype × sex interaction effect measured at the network level. AU, auditory; CE, cerebellar; CO, cingulo-opercular task control; DM, default mode; DA, dorsal attention, FP, fronto-parietal task control; ME, memory retrieval; SA, salience; SML, lateral sensorimotor; SMM, medial sensorimotor; SC, subcortical; VA, ventral attention; VI, visual.

We detected significant genotype × sex interaction effects on network activation related to intertemporal decision-making for the DM, SC, AU, SMM, CO, DA, and CE networks (Figure 3A). The strength and direction of this relationship depended on the strength and direction of task-related neural activation. To quantify this relationship between task-related activation and *COMT* genotype × sex interaction effects, beta estimates of the genotype × sex interaction effect on task-related activation were plotted against the corresponding beta estimates of task-related activation for each ROI; a Pearson correlation analysis indicated a significant positive correlation (*r* = 0.69, *p* < 0.001; Figure 3B) in which greater positive task-related activation predicted a stronger U-shaped relationship with *COMT* genotype, whereas more negative task-related activation predicted an inverted U-shaped relationship with *COMT* genotype.

### Resting-state functional connectivity

**Figure 4:**
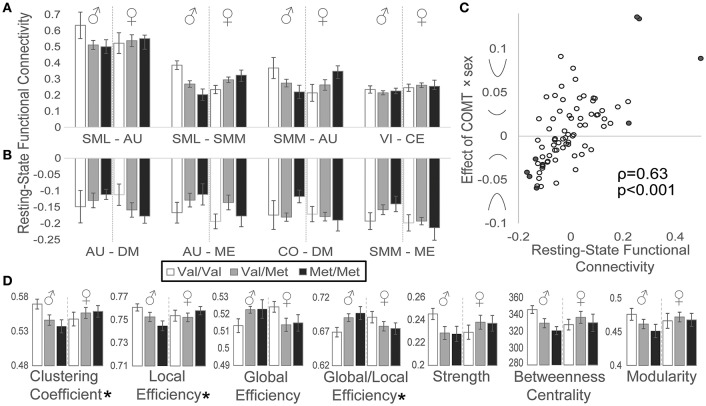
Effects of *COMT* genotype and sex on functional connectivity during a resting state. Between-network functional connectivity is plotted by *COMT* genotype and sex for the **(A)** four most positive and **(B)** four most negative connections. For display purposes, between-network connectivity was plotted as the averaged Fisher-z-transformed correlation between all ROIs belonging to each pair of networks. Error bars reflect standard errors of the means. **(C)** Scatter plot of resting-state functional connectivity plotted against *COMT* genotype × sex interaction effects for each network pair. Symbols on the y-axis correspond with the direction of interaction effects, producing u-shaped (positive values) or inverted u-shaped (negative values) relationships. Filled circles indicate network connections highlighted in panels A and B. **(D)** Graph theory measures are plotted by *COMT* genotype and sex. (^*^) Denotes statistically significant *COMT* genotype × sex interaction effect measured at the network level. Error bars reflect standard errors of the means. AU, auditory; CE, cerebellar; CO, cingulo-opercular task control; DM, default mode; ME, memory retrieval; SML, lateral sensorimotor; SMM, medial sensorimotor; VI, visual.

We also detected effects of *COMT* genotype on functional connectivity during rest. Network connectivity by genotype and sex is plotted for the four most positively (Figure 4A) and most negatively (Figure 4B) correlated network pairs during the resting state. As observed for the task-based activation data, we similarly detected a relationship between resting-state functional connectivity and the strength and direction of *COMT* genotype-by-sex effects (Figure 4C): greater positive functional connectivity associated with stronger COMT genotype × sex U-shaped effects, whereas more negative functional connectivity associated with inverted U-shaped relationships based on Spearman correlation analysis (ρ = 0.63, *p* < 0.001).

In addition to measuring simple correlations between network time series, we also evaluated effects of sex and *COMT* genotype on several graph theory metrics (Figure 4D). We observed significant genotype × sex interaction effects, reflecting U-shaped relationships, on both the clustering coefficient (*t* = 2.00, *p* = 0.049) and local efficiency (*t* = 2.01, *p* = 0.048), which both measure aspects of how nodes cluster together. Conversely, *COMT* effects on global efficiency, the inverse of the average shortest path length between all nodes, demonstrated a trend toward an inverted U-shaped curve, although this effect did not reach statistical significance (*t* = −1.86, *p* = 0.067). However, the tendency toward favoring global over local efficiency, represented by the ratio of global efficiency/local efficiency, exhibited a significant inverted U-shaped relationship (*t* = −2.30, *p* = 0.024). We failed to detect significant effects of *COMT* genotype and/or sex on functional connectivity strength, betweenness centrality, or modularity.

### Functional connectivity flexibility

**Figure 5:**
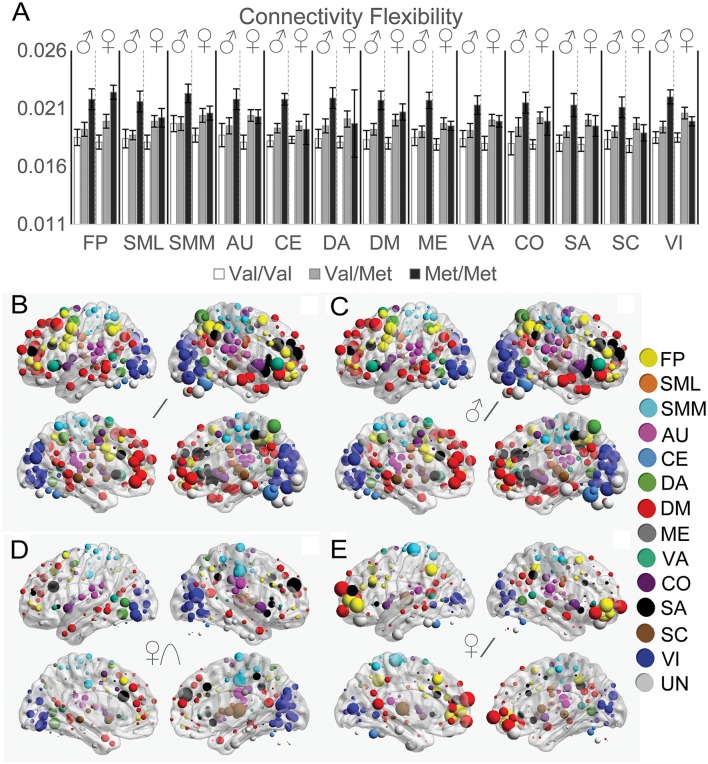
Effects of *COMT* genotype and sex on functional connectivity flexibility between resting and task states. **(A)** Functional connectivity flexibility is plotted by *COMT* genotype and sex for each large-scale network. For display purposes, connectivity flexibility was averaged across all ROIs in a given network. Error bars reflect standard errors of the means. The strength of *COMT* genotype effects for each of the 264 ROIs is displayed on left and right cerebral hemispheres according to statistical tests of **(B)** linear effects across all subjects, controlling for sex, **(C)** linear effects in males, **(D)** quadratic effects in females, and **(E)** linear effects in females. The size of the spheres correspond with the size of the *COMT* genotype effects, scaled independently for each of the image sets in **(B–E)**. AU, auditory; CE, cerebellar; CO, cingulo-opercular task control; DM, default mode; DA, dorsal attention, FP, fronto-parietal task control; ME, memory retrieval; SA, salience; SML, lateral sensorimotor; SMM, medial sensorimotor; SC, subcortical; VA, ventral attention; VI, visual.

To quantify how much functional connectivity patterns changed across different states, we next investigated *COMT* genotype and sex effects on the distance between functional connectivity matrices across resting and task states (Figure 5). For the FP, SML, and SMM networks, we observed a linear effect of *COMT* that did not significantly differ between the sexes (Figure 5A). However, we found significant genotype × sex interaction effects on multiple networks: AU, CE, CO, DA, DM, ME, SA, SC, VA, and VI. Whereas effects of *COMT* genotype exhibited linear effects on data from males for all networks, for females, Akaike Information Criteria indicated an improved fit of quadratic vs. linear relationships for the CO, SA, SC, and VI networks. A linear relationship best described the effect of *COMT* genotype on the FP, SML, SMM, AU, CE, DA, DM, ME, and VA networks among females. In Figure 5, the strength of *COMT* genotype effects for each of the 264 ROIs is depicted for all subjects (Figure 5B), linear effects in males (Figure 5C), quadratic effects in females (Figure 5D), and linear effects in females (Figure 5E). The observed *COMT* effects on functional connectivity flexibility did not appear to be driven by functional connectivity changes associated with any one brain state (Supplementary Figure 1).

## Discussion

**Figure 6:**
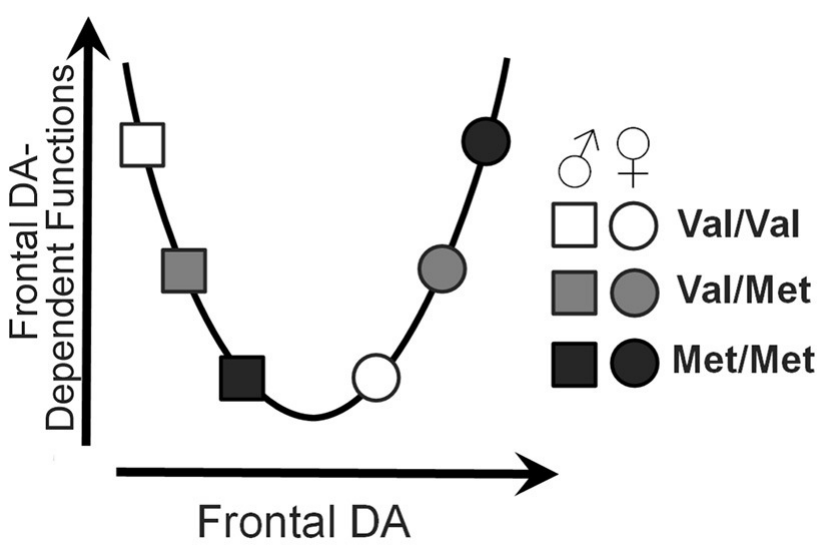
U-shaped model of frontal dopamine function. Theoretical influences of both sex and *COMT* genotype on frontal dopamine levels by which sex and *COMT* genotype may affect frontal dopamine-dependent functions.

We investigated the role of the Val^158^Met polymorphism of the *COMT* gene on several properties of neural function. Measures of task-evoked activity and resting-state functional connections demonstrated significant genotype × sex interactions, resulting in U-shaped or inverted U-shaped relationships with COMT enzyme activity; the direction and extent of these genotype effects depended on the direction and extent of the activity or connectivity. The findings are largely consistent with the extensive literature demonstrating U-shaped effects of *COMT* genotype and theoretical frontal dopamine levels on executive function. A model describing the theoretical relationship between sex, genotype, and executive functions based on the data relationships observed in the current study is presented in Figure 6.

The neural effects of *COMT* genotype observed in this study were evident in all networks that we tested and appear to promote brain-wide alterations in neural activity and connectivity. Although, COMT activity is thought to affect dopamine signaling principally in the frontal cortex through alterations in synaptic clearance rates of dopamine, evidence from rodent studies also supports an association of COMT activity with dopamine release in the striatum (Simpson et al., 2014) and hippocampus (Laatikainen et al., 2013), and with dopamine metabolism throughout the brain (Laatikainen et al., 2013). Although some effects may be mediated through frontal dopamine signaling in functionally-connected brain regions, a previous task-activation study notably identified effects of *COMT Val*^158^*Met* genotype only for posterior default mode deactivation (Malhotra et al., 2002).

In fact, our findings demonstrate that the effects of *COMT* genotype on fMRI blood oxygen level-dependent (BOLD) signal are most pronounced in regions that are modulated most during a delay discounting task and, additionally, among those connections that are strongest during the resting state. A possible explanation for these findings come from studies in rats showing that a COMT inhibitor did not affect basal dopamine levels but instead enhanced extracellular dopamine levels in response to elicited dopamine release (Tunbridge et al., 2004). Thus, an inference from the current findings is that the observed *COMT* genotype-by-sex effects may be driven by task-evoked dopamine release in the associated regions/networks. Although a role for norepinephrine cannot be excluded, most evidence to-date has emphasized a dominant role for the COMT enzyme in preferentially modulating dopamine activity in the brain (Gogos et al., 1998; Tunbridge et al., 2004; Laatikainen et al., 2013).

The effects of *COMT* genotype on the resting state extended to influences on brain organization as indexed by graph theoretical measures. In particular, there were significant genotype × sex interactions on the clustering coefficient and local efficiency. The clustering coefficient indexes the extent to which a node's neighbors (i.e., the other nodes to which it is connected) are also connected to each other. A related measure, local efficiency is the inverse of the average shortest path length connecting a node's neighbors. Interestingly, the observed effects of *COMT* genotype on these measures suggest that very low or very high dopamine enhances the clustering and local efficiency throughout the brain. Combining these findings with the observation that the ratio of global-to-local efficiency had an inverted U-shaped relationship to putative COMT activity suggest that intermediate levels of COMT activity—and perhaps, therefore, intermediate levels of dopamine—promote greater global efficiency at the expense of local efficiency, whereas potentially suboptimal levels of dopamine (either very high or very low) favor localized clustering and local efficiency.

In this study, we introduce a novel metric of functional connectivity flexibility, calculated as the average Euclidean distance between functional connectivity matrices across multiple brain states, which indicates the extent to which connections between regions are reorganized in response to state changes. For many regions, both males and females exhibited a positive linear relationship with *COMT* genotype (i.e., *Val*/*Val* < *Val*/*Met* < *Met*/*Met*), while a subset of regions/networks exhibit a quadratic relationship among females. These findings are seemingly in contrast with behavioral evidence that the *Val*/*Val* genotype is associated with higher cognitive flexibility in a task-switching paradigm (Colzato et al., 2010). One interpretation is that *Met*/*Met* individuals must undergo more extensive neural reconfiguration to produce behavioral changes, leading to decreased efficiency. Another possibility is that an increased signal-to-noise ratio in fMRI data associated with the *Met*/*Met* genotype (Winterer et al., 2006) enhanced the detection of functional connections in both the positive and negative directions, producing larger flexibility scores.

Notably, there was not an apparent U-shaped relationship between the flexibility of functional connectivity and theoretical dopamine levels. One possible explanation is that very high levels of dopamine do not impair functional connectivity flexibility, producing a mostly linear relationship. This interpretation would receive support if females, with higher dopamine levels, demonstrated increased flexibility relative to males, which was not the case. Another possibility is that these effects are primarily driven by factors other than dopamine. One such possible factor is blood pressure, since COMT genotype has been linked to variations in blood pressure among men (Annerbrink et al., 2008), likely due to differences in peripheral catecholamine metabolism, and blood pressure influences the BOLD signal (Kalisch et al., 2001).

Although the current consensus is that the *COMT Val*^158^*Met* polymorphism does not play a dominant role in most mental health disorders (Hosak, 2007; Klein et al., 2016), the pleiotropic influence of this gene on intermediate phenotypes of several psychiatric disorders has drawn considerable interest. For example, a prominent effect of *COMT* genotype was found for delay discounting-related activation of the default-mode network, known to support future-oriented thinking (Spreng et al., 2009), highlighting a potential neural mechanism by which *COMT* may modulate this intermediate phenotype of addiction. Additionally, modulation of COMT activity by sex may contribute to sex differences in many psychiatric disorders (Harrison and Tunbridge, 2008). In fact, the current data suggest that the original “worrier-vs.-warrior” conceptualization (Stein et al., 2006) in which *Val* alleles confer protection from anxiety (“warriors”) whereas *Met* alleles offer advantages for attention (“worriers”) may need to be refined. Rather, males (particularly *Val*/*Val*) may be more likely to exhibit a “warrior” phenotype, and females (particularly *Met*/*Met*) may be more likely to characterize the “worrier” phenotype; as such, male and female sex may generally be protective against the “worrier” phenotype and “warrior” phenotype, respectively. This distinction is consistent with the fact that males are overrepresented within “externalizing disorders” like ADHD and antisocial personality, whereas females are more prone to “internalizing disorders” such as anxiety and depression.

### Limitations

Although *COMT* genotype is closely related to COMT activity and therefore to frontal dopamine levels, there is not a perfect correspondence between these measures. Future studies that evaluate effects of more direct manipulation of dopamine levels are warranted. Studies of *COMT* methylation could also account for variance in COMT activity related to epigenetic modifications. Furthermore, we did not measure blood pressure or other physiological measures, so we could not account for potential peripheral effects of *COMT* genotype. Moreover, despite the seemingly robust effects of this polymorphism, some cell sizes were relatively small (e.g., 8 Val/Val females), limiting confidence in the stability of the findings. Finally, the generalizability of these findings to other tasks remains to be tested.

## Conclusion

We report linear and U-shaped relationships associated with a *COMT* polymorphism and sex with fMRI measures of neural function. Our results indicate brain-wide effects of *COMT* genotype, indicating potentially broader effects of *COMT* genotype beyond localized effects on frontal dopamine dynamics.

## Ethics statement

This study was carried out in accordance with the recommendations of the Declaration of Helsinki of the World Medical Association (WMA) with written informed consent from all subjects. All subjects gave written informed consent in accordance with the Declaration of Helsinki. The protocol was approved by the University of North Carolina, Chapel Hill (UNC) Office of Human Research Ethics.

## Author contributions

CB and CS were responsible for the study concept and design. CS and MP contributed to the acquisition of data and assisted with behavioral data analysis. AE assisted with behavioral analysis and performed the fMRI data analysis. AE and CB drafted the manuscript. All authors critically reviewed content and approved final version for publication.

### Conflict of interest statement

The authors declare that the research was conducted in the absence of any commercial or financial relationships that could be construed as a potential conflict of interest.
