# Supplementary material for: COMT Val158Met Polymorphism Exerts Sex-Dependent Effects on fMRI Measures of Brain Function
Source: Front Hum Neurosci. 2017 Dec 6;11:578. doi: 10.3389/fnhum.2017.00578 (PMC5723646; doi:10.3389/fnhum.2017.00578)
Supplement: Supplementary file 1 [file Image1.pdf]

Supplementary Material

Delay discounting task

While in the MRI scanner, participants performed a delay discounting task optimized for fMRI studies[3, 35]. Following a short training task, subjects underwent six consecutive functional MRI scans (~8 minutes per scan) each of which included 32 *Now* versus *Later* decisions. An instruction cue, presented for 4.4 seconds, preceded the *Now* and *Later* options to indicate one of four choice trial types, pseudorandomly determined: "WANT," "DON'T WANT," "SOONER," or "LARGER". Using an MRI-compatible button box, subjects selected their preferred option in WANT trials (~99 trials total) and their non-preferred option in DON'T WANT trials (~31 trials total). In SOONER trials (~31 trials total), participants objectively identified the option available sooner in time, whereas they identified the objectively larger reward in LARGER trials (~31 trials total). *Later* options ($2, $5, $10, $20, or $100) were offered at one of five future delays: 1 week, 2 weeks, 1 month, 3 months, or 6 months. The *Now* option, available "Today," represented a 30%, 15%, 10%, or 5% reduction from the *Later* amount. Delays, reward values, discounts, and left/right position were randomized across trials. *Now* and *Later* options were visible on the screen for 4.4 seconds. During "null" trials (30 trials total), an instruction cue was presented but no *Now* and *Later* choice options were presented. Inter-trial intervals were jittered between 4.4-8.8 seconds.

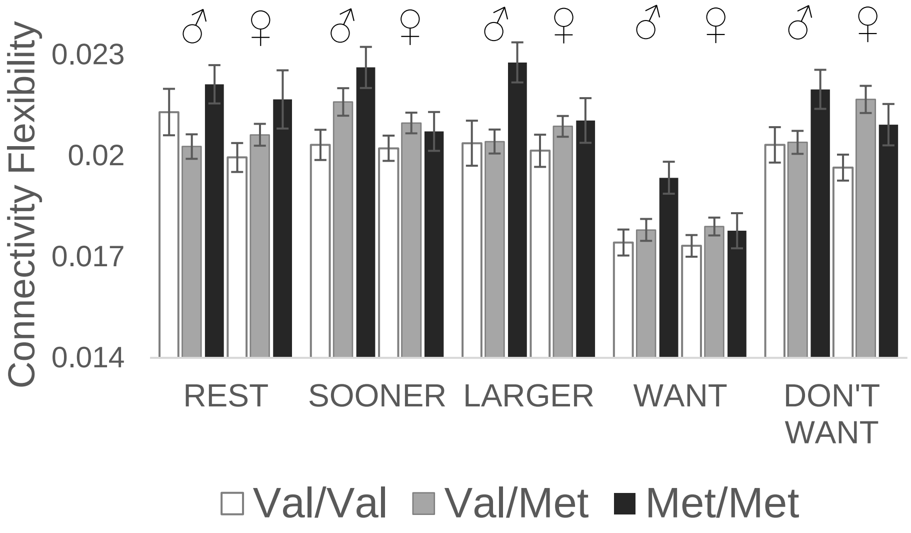

Supplementary Figure 1. Functional connectivity flexibility is plotted for each rest/task state, for each genotype and sex, averaged over all 264 ROIs. Following calculation of functional connectivity matrices for each state, functional connectivity flexibility was calculated as the average Euclidean distance between a given state and each of the four other states. Larger connectivity flexibility values indicate that functional connectivity during a given state demonstrates greater changes from that of other states.
